# Optimizing performance and yield of vertical GaN diodes using wafer scale optical techniques

**DOI:** 10.1038/s41598-021-04170-2

**Published:** 2022-01-13

**Authors:** James C. Gallagher, Mona A. Ebrish, Matthew A. Porter, Alan G. Jacobs, Brendan P. Gunning, Robert J. Kaplar, Karl D. Hobart, Travis J. Anderson

**Affiliations:** 1 U.S. Naval Research Laboratory, 4555 Overlook Ave SW, Washington, DC 20375 USA; 2 NRC Postdoc Fellow Residing at the U.S. Naval Research Laboratory, Washington, DC 20375, USA; 3 Naval Postgraduate School, 1 University Dr, Monterey, CA 93943 USA; 4 Sandia National Laboratories, MS 1086, PO Box 5800, Albuquerque, NM 87185 USA

**Keywords:** Electronic devices, Semiconductors, Electrical and electronic engineering

## Abstract

To improve the manufacturing of vertical GaN devices for power electronics applications, the effects of defects in GaN substrates need to be better understood. Many non-destructive techniques including photoluminescence, Raman spectroscopy and optical profilometry, can be used to detect defects in the substrate and epitaxial layers. Raman spectroscopy was used to identify points of high crystal stress and non-uniform conductivity in a substrate, while optical profilometry was used to identify bumps and pits in a substrate which could cause catastrophic device failures. The effect of the defects was studied using vertical P-i-N diodes with a single zone junction termination extention (JTE) edge termination and isolation, which were formed via nitrogen implantation. Diodes were fabricated on and off of sample abnormalities to study their effects. From electrical measurements, it was discovered that the devices could consistently block voltages over 1000 V (near the theoretical value of the epitaxial layer design), and the forward bias behavior could consistently produce on-resistance below 2 mΩ cm^2^, which is an excellent value considering DC biasing was used and no substrate thinning was performed. It was found that high crystal stress increased the probability of device failure from 6 to 20%, while an inhomogeneous carrier concentration had little effect on reverse bias behavior, and slightly (~ 3%) increased the on-resistance (R_on_). Optical profilometry was able to detect regions of high surface roughness, bumps, and pits; in which, the majority of the defects detected were benign. However a large bump in the termination region of the JTE or a deep pit can induce a low voltage catastrophic failure, and increased crystal stress detected by the Raman correlated to the optical profilometry with associated surface topography.

## Introduction

There has been significant research interest to improve power electronic technology using GaN. Currently the majority of power electronic devices are Si based, with SiC expected to be the industry standand in the near future^1^. The lower on-resistance (R_on_) and higher breakdown voltage of GaN could enable higher voltage devices^2,3^ and smaller devices at higher frequencies^4,5^. This technology could be realized by a reliable vertical GaN manufacturing process. GaN substrate manufacturing technology, however, is still immature,^4,6–8^ and thus heteroepitaxial lateral AlGaN/GaN High Mobility Electron Transistor (HEMT) technology is the industry standard for low voltage devices., Large die sizes are required to produce a breakdown voltage above a kilovolt in lateral GaN devices due to reduced critical field in such device designs^4,5,9–11^. A switch to vertical geometry would increase critical electric field by a factor of 3, improve the breakdown voltage by an order of magnitude, and increase the resistance to radiation damage^4,5,12–15^.

Though 2’’ GaN substrates are readily available, their properties are inconsistent. A major road block in vertical GaN device manufacturing is finding a wafer scale method for screening. Many non-destructive techniques including photoluminescence, x-ray topography, optical profilometry, and Raman spectroscopy mapping can be used to detect defects in the substrates and epitaxial layers^12,16,17^; however, it is important to determine how much these defects affect the diode properties. In our previous research^17^, we tested the effects of high dislocation density caused by crystal stress points on trench isolated diode performance, showing that an electrically uniform substrate as detected by Raman produces better quality diodes. This work exploits the use of both optical profilometry and Raman spectroscopy to predict the quality of diodes with junction termination extenstions (JTEs). The JTE design reduces the potential for sidewall leakage and regions with high electric field. This allows for higher voltage testing, which is essential to high power device manufacturing.

## Experimental details

### Sample fabrication

**Figure 1:**
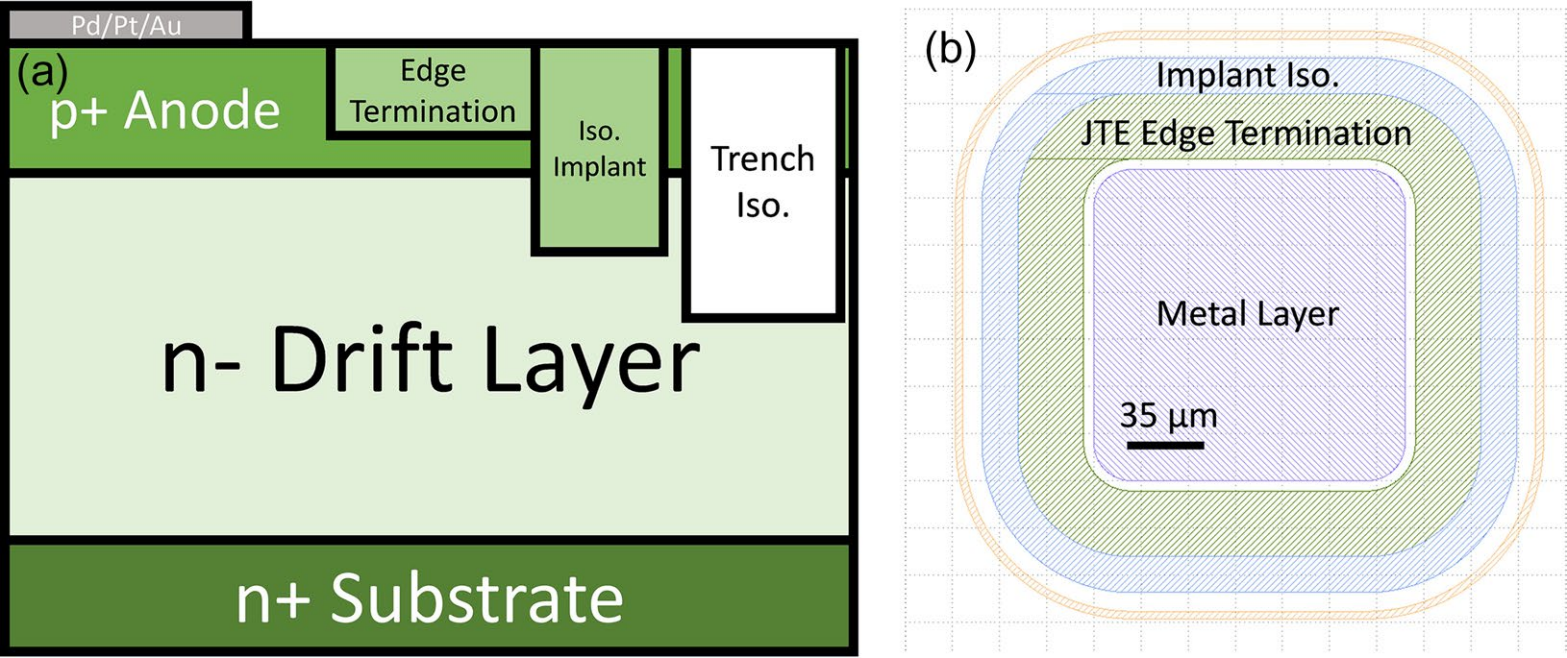
Side view (**a**) and top view (**b**) diagrams of the vertical PN diodes fabricated for this study.

**Figure 2:**
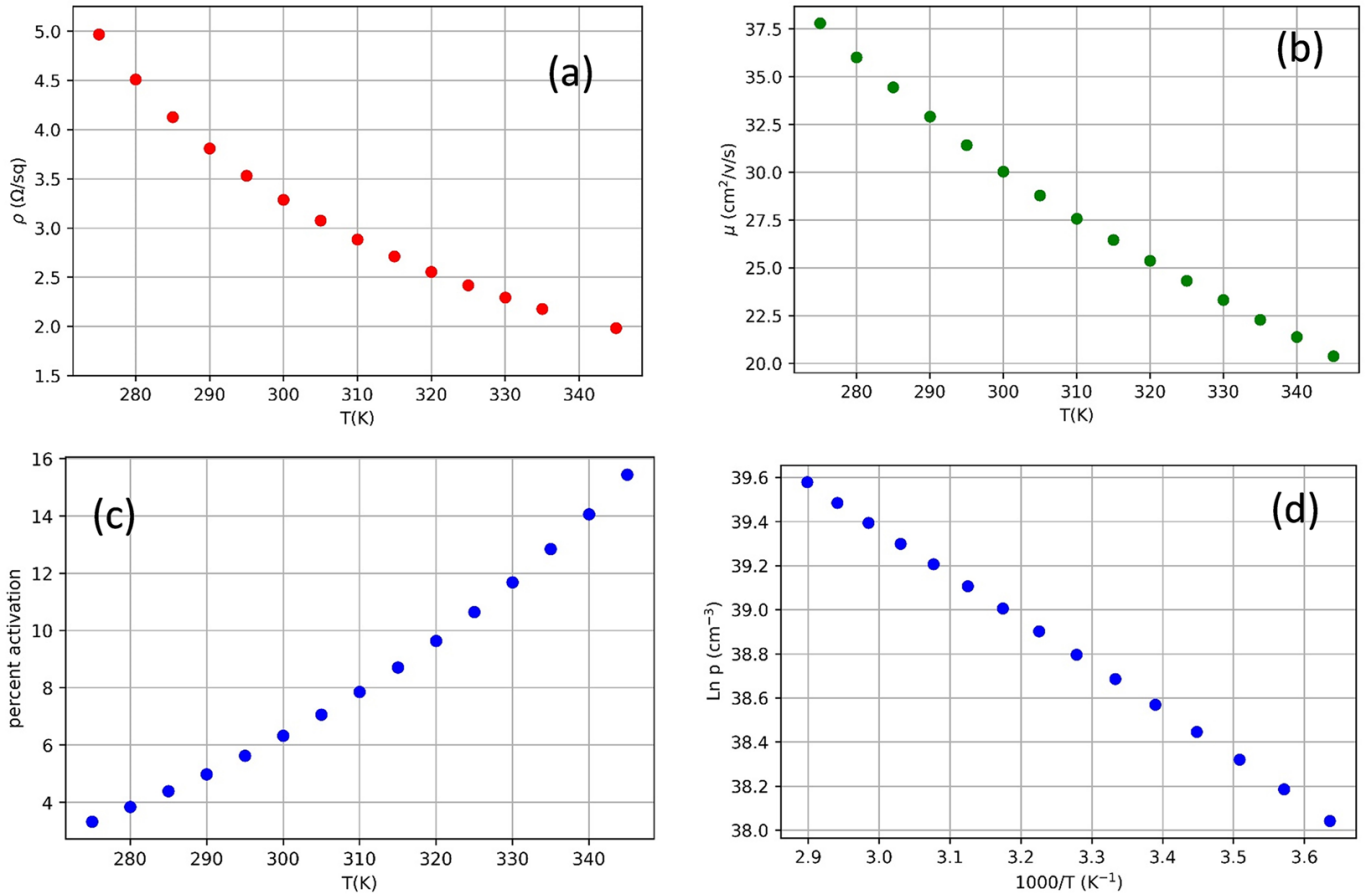
The resistance (**a**), hole mobility (**b**), and the hole concentration (**c**–**d**) are taken as a function at temperature ranging from 275 to 345 K. The hole concentration is in units of percentrage of the Mg atoms activated with 100% activation = 10^18^ cm^−3^. (**d**) The hole concentration is plotted on *Ln(p)* vs 1000/*T* scale to show lineararity.

Vertical P-i-N GaN epilayers were fabricated by MOCVD in a Taiyo Nippon Sanso SR4000HT reactor. The n-type layer was a type IIa substrate with a periodic structure, which is an artifact of the manufacturing technique, similar to our previous work^17^. The 8 µm drift layer was Si doped n-type with n ≈ 2E16 cm^−3^. The 500 nm p-type layer was doped with [Mg] = 1E18 cm^−3^. Vertical diodes were fabricated on these layers using a Cl_2_ plasma etch to create trench isolation, a multi-energy nitrogen implant profile approximating a box was used to create a ~ 600 nm deep implant isolation inside the trench, and a ~ 300 nm deep nitrogen implant was used for a single zone JTE edge termination. Implanted structures were utilized as-implanted without subsequent annealing and only subject to low processing induced temperatures not exceeding 300 °C. Ohmic contacts were deposited using Pd/Pt/Au on the p-layer and Ti/Al/Ni/Au on the substrate. The sample device diagram is shown in Fig. 1. The activation percentage of the p-layer was studied using a Lakeshore cryogenic Hall effect system with a M91 FastHall™ measurement controller. At room temperature, the hole concentration was found to be 6.3 × 10^16^ cm^−3^ giving a 6.3% activation. Using temperature dependent Hall effect data taken from 275 to 345 K (see Fig. 2.), the hole concentration (*p*) was fit to

1 $$p = Ae^{{{\raise0.7ex\hbox{${ - E_{A} }$} \!\mathord{\left/ {\vphantom {{ - E_{A} } {k_{b} T}}}\right.\kern-\nulldelimiterspace} \!\lower0.7ex\hbox{${k_{b} T}$}}}}$$

(from Eq. 2.50 in reference^18^). The activation energy (*E*_*A*_) was determined to be 177 meV, in agreement with the literature results^19,20^. Six grids (labeled a–f see supplemental materials) containing 49 devies each in a 7 × 7 grid were fabricated for a total of 294 devices. The grids were aligned using Raman spectroscopy, discussed in the next section.

### Optical measurements

**Figure 3:**
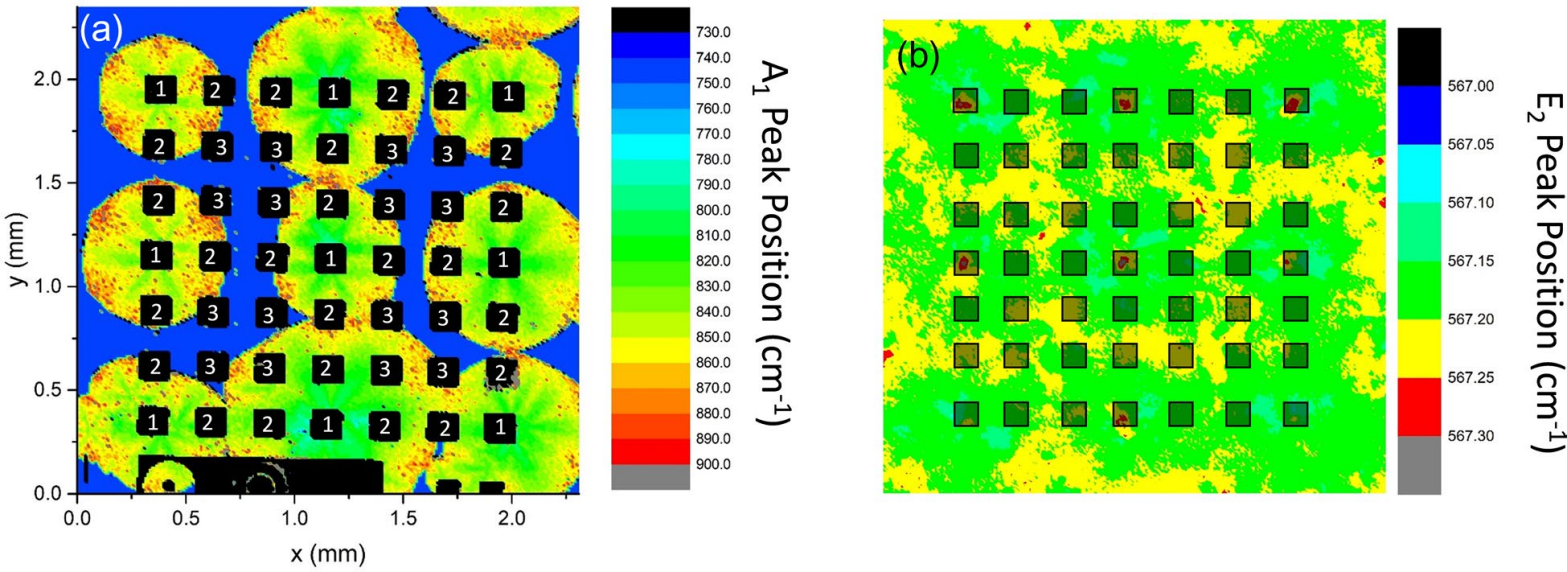
(**a**) Raman map of the A_1_ (LO) peak on a 7 × 7 device reticle; the black areas are the regions are the metal contact to the anode. (**b**) Raman map of the E_2_ peak of a similar region. Translucent black boxes are drawn where the diodes are expected to be fabricated. The devices are divided into 3 positons labeled in (**a**): Postion 1, on a point of high crystal stress; Position 2, adjacent to point of high crystal stress thus still on a point of higher carrier concentration; Position 3, on a higher resistivity point away from the crystal stress points.

In previous work^12,17,21–23^, it was shown that Raman spectroscopy is effective at detecting changes in carrier concentration and crystal stress in the susbtrate. When using the 532 nm laser with 10 × objective and depth of focus of approximately 100 µm, the spectra primarily probes the substrate due to the transparency of GaN. This technique can a full 2 inch wafer with 20 µm resolution in 1–3 days depending on the acceptable noise level. Some GaN wafers, such as those grown using the dot-core technique^24^, have a regular pattern of defects. The wafer used in this study has periodic points of crystal stress (detected by a shift in the Raman E_2_ peak^25^). These crystal stress points an increase the carrier concentration in a hexagonal structure detected by the shifting of the A_1_ (LO) peak. Using Raman, the diodes were aligned in 7 × 7 grids such that the corner points were on the high crystal stress points as shown in Fig. 3. The devices in Fig. 3a are numbered to represent their position relative to the high crystal stress points and the high resistivity regions. Position 1 includes devices on the points of high crystal stress, and are on the most conductive part of the substrate. Position 2 are adjacent (133 µm) to the points of high crystal stress. Position 3 covers the devices diagonally (188 µm) from the points of crystal stress thus cover the less conductive regions of the substrate.

**Figure 4:**
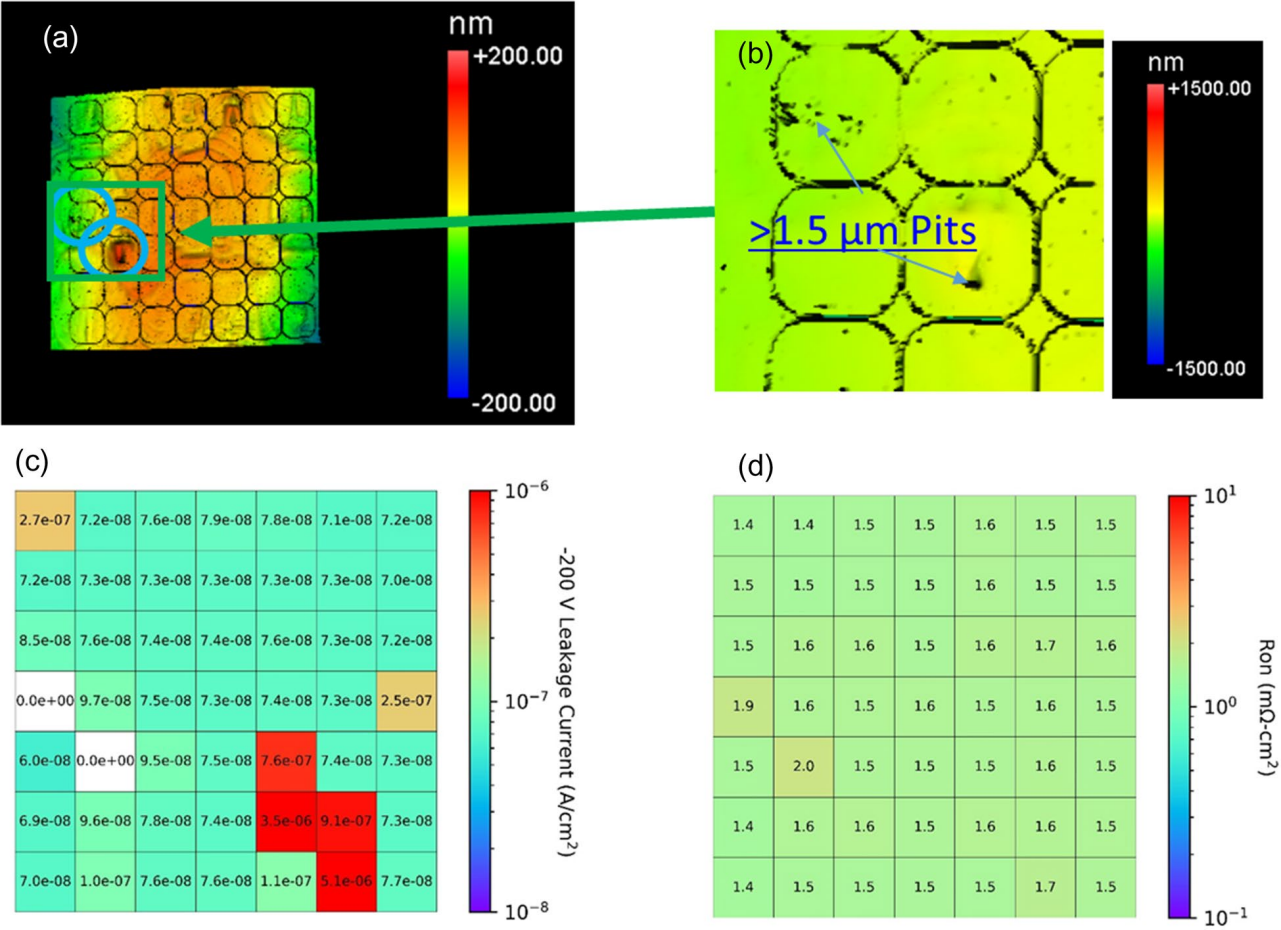
(**a**) Representative (grid d) optical profilimietry map is shown. (**b**) Is an inset of of the green rectangle in (**a**) revealing deap 1.5 µm pits. (**c**) And (**d**) are corresponding maps of the leakage current at − 200 V and the forward on resistance respectively. The white devices catastrophically failed before reaching − 200 V. The data for the other grids can be found in the supplemental materials.

The regions were also studied using a Zygo™ NewView 7300 optical profilimeter with a 25 × magnification giving an x–y resolution of 442 nm/pixel. This technique is capable of mapping a full 2 inch wafer in a few hours. The scans were taken after the Cl_2_ trench isolation etch, but before all other device processing steps. Figure 4a shows the optical profilometry on grid d (see Figure S1 in supplemental materials for information about the grids). The results show that the high crystal stress points (position 1) may manifest themselves as abnormal areas on the surface in the Zygo scan. Other surface defects can be detected; however, non-uniform conductivity has no clear effect on the surface morphology besides features coinciding with those due to high stress observed via Raman imaging.

### Electrical measurements

**Figure 5:**
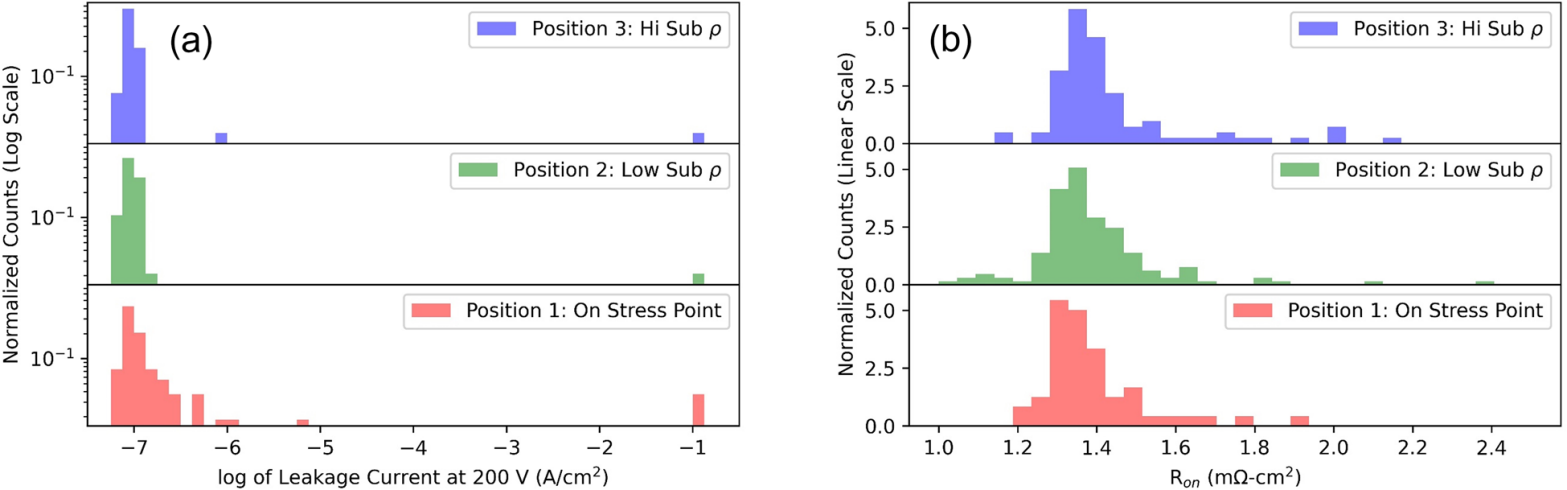
A histogram distribution of reverse leakage currents (**a**) and on resistance (**b**). The data is separated based on the 3 regions defined in Fig. 3a. Catostrophic failures were set at the saturation current of the instrument (100 mA) in part (**a**).

**Figure 6:**
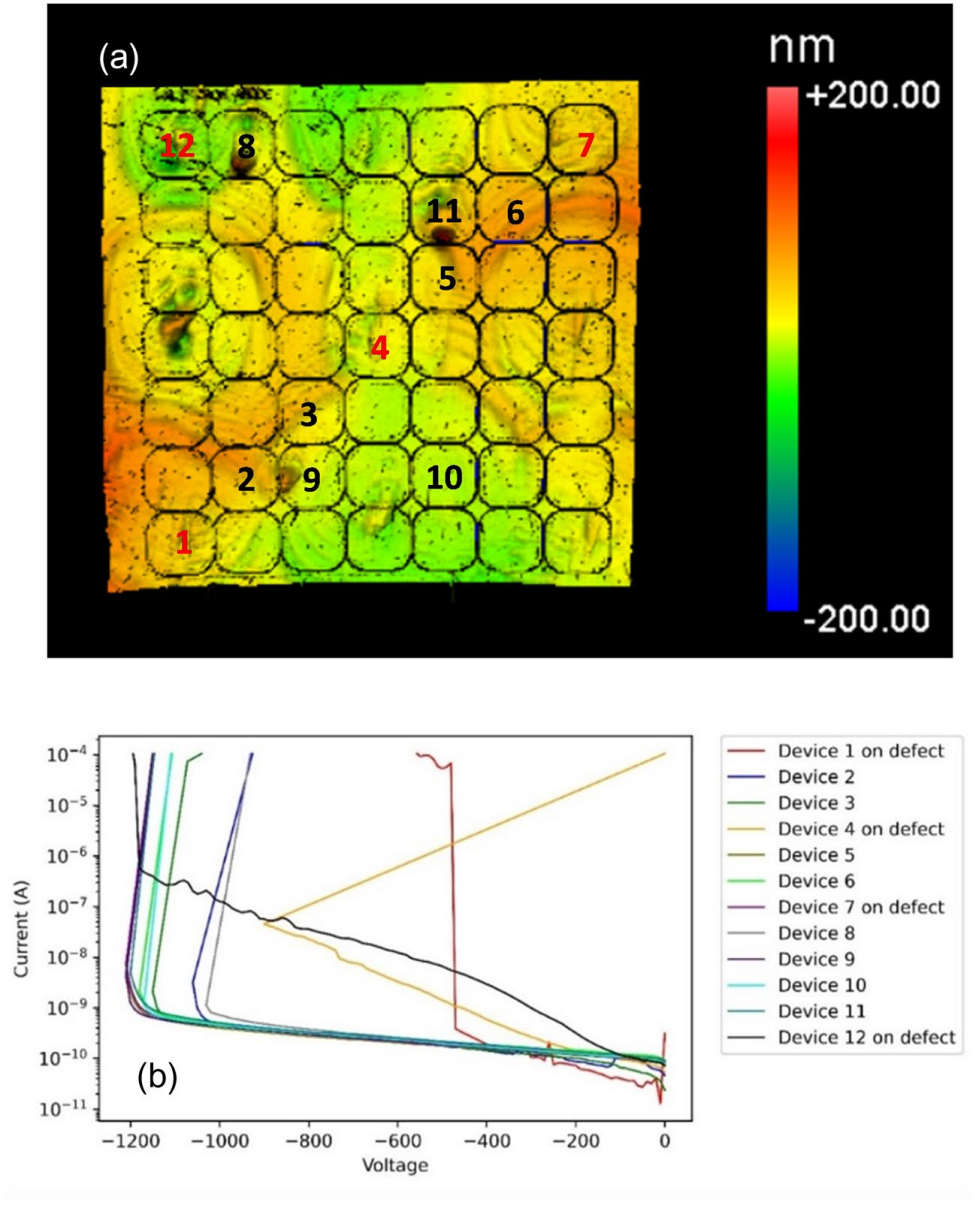
(**a**) Optical profilometry image of grid A is shown. Devices with red number are on point of high crystal stress. The devices tested with breakdown IV curves (**b**) are labed.

Electrical measurements were taken on all devices with a Keithley 4200 with an SMU pre-amplifier. All samples were measured from − 10 to 10 V on the anode, followed by a intial blocking test to − 200 V. Figures 4c, d show − 200 V example reverse leakage currents and and specific on resistance, respectively, for a single reticle. Additional data are avalible in the supplemental materials. Histogram plots of the distribution of the data is shown in Fig. 5. High voltage breakdown curves were taken on 12 devices in grid A using the Keithly 2657A. The results are shown in Fig. 6.

### Simulations

**Figure 7:**
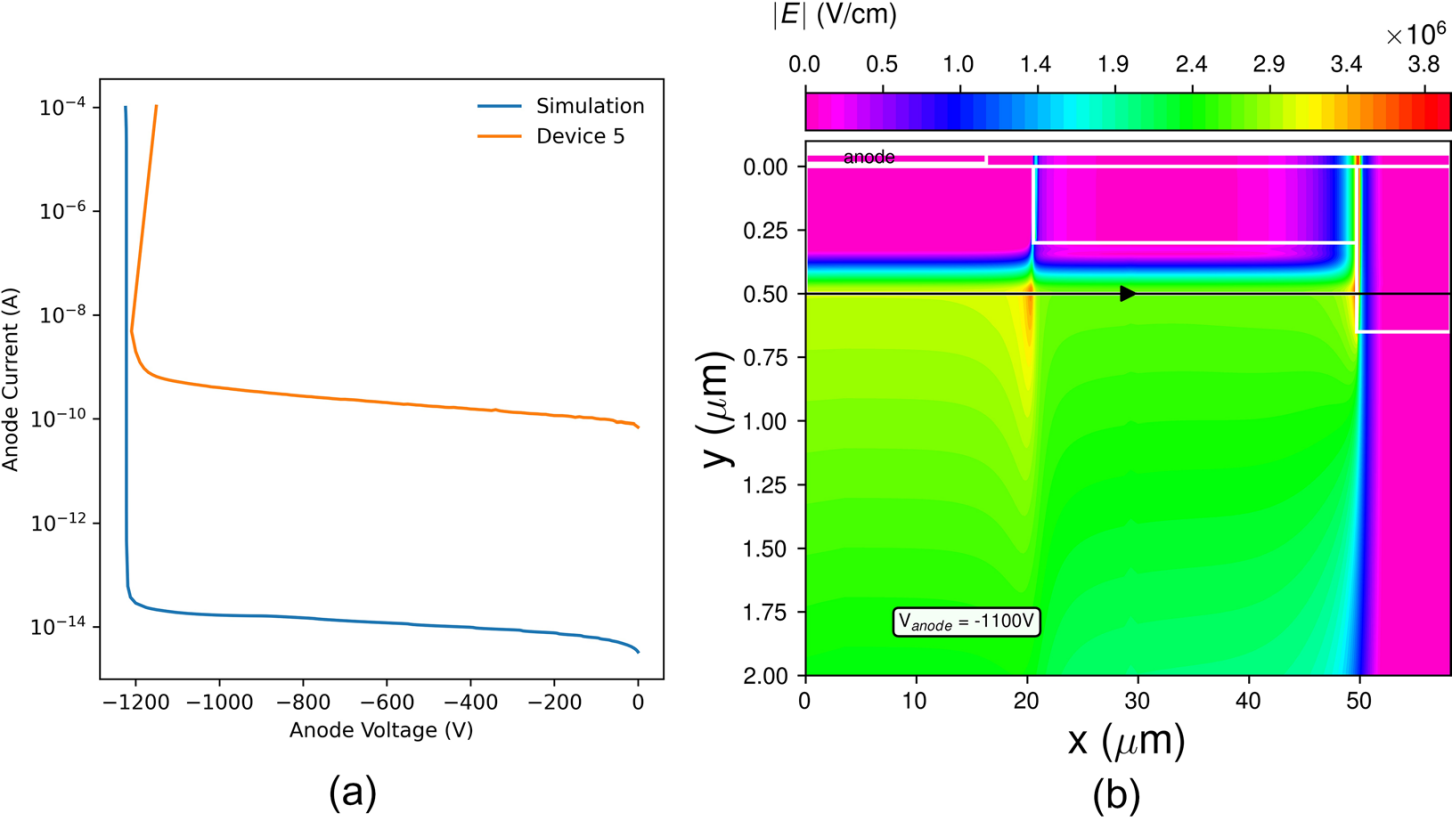
Results of simulations of reverse breakdown of the defect-free device under study. (**a**) shows a comparison between the simulated and measured reverse IV characteristics of a representative defect-free device. A contour plot of the electric field in the edge termination region at an anode bias of − 1200 V is shown in (**b**).

To explore the predicted upper bound of breakdown voltage in devices with the edge termination structure illustrated in Fig. 1, simulations of device breakdown were carried out using Silvaco ATLAS. The geometry of the simulated area was decided by the radius of the edge termination region in the corners of the device area, based upon the assumption that the worst case electric field magnitudes would occur there. Accordingly, the device is simulated with cylindrical symmetry about the left-side axis. To accurately simulate the edge termination region, the TRIM Monte Carlo simulator was used to determine the expected profile of vacancies and interstitials resulting from N implants of 25, 35, 135 and 150 keV into GaN. These profiles were implemented in the device simulation as mid-gap trap states, per theoretical considerations in^26^. Impact ionization was assumed to be a local process, and a fit to a Chynoweth model using experimental data from^27^ and was used to implement the field dependence of the impact ionization coefficients. Results from the simulations are shown in Fig. 7.

## Discussion

This result reveals that this GaN growth and device probing technique produces high quality results. Most devices had an ideality factor close to 2 and an on-resistance below 2 mΩ cm^2^. The majority of devices could block 200 V reverse bias and many had breakdown voltage above 1000 V. These are good results for the thickness of the epitaxial layers making these good samples for analyzing the effects of the long-range non destructive techniques.

Dividing the reverse bias electrical data spectroscopy results into the 3 different positions defined by the Raman spectroscopy (see Fig. 3) reveals much about the substrate quality and the effect of said quality on the diode. The results are summarized in Table 1. From the histogram plot in Fig. 5a it is revealed that the devices in position 3 have the highest percentage of devices with reverse leakage current below 1 × 10^–7^ (A/cm^2^). In contrast, the devices in position 1 have a large percentage (See Table 1) with higher leakage current. However, the JTE design does mitigate the effects of the high crystal stress points. MESA isolated diodes from our previous research^17^ show that using a simple MESA isolated structure without edge termination consistently produces leakage currents above 10 mA/cm^2^ at 200 V, while in this study we found 80% of the devices even on points of high stress produced leakage currents below 2 × 10^–7^ A/cm^2^.

**Table 1:** A summary of the electrical results taken in the 3 different Raman positions defined in Fig. 3a. The data used in the analysis is shown in Figures S2 and S3.

Position	Number of devices	Number of catostrophic failures < 200 V	High leakage fail (> 1E–7 A/cm^2^)	High leakage fail (> 2E–7 A/cm^2^)	Median J (A/cm^2^) at − 200 V	Median R_on_ (mΩ-cm^2^)
1	54	1	50%	20%	1.01E−7	1.34
2	144	3	37%	5%	9.1E−8	1.36
3	96	2	29%	6%	9.2E−8	1.38
All	296	6	37%	9%	9.2E−8	1.37

Mapping results of Raman imaging versus electrical characteristics, devices in positions 2 and 3 typically produce better results than those in position 1. This is likely because the crystal stress points translate into defects in the epitaxial layer as can be observed by optical profilometry; however, inhomogeneous conductivity does not manifest itself on the surface thus the epi layers remain high quality. This is more apparent at higher voltages. Figure 6 shows the reverse characteristics of 12 diodes to breakdown. All devices tested in postions 2 or 3 had a breakdown voltage above 1000 V. Of the four diodes tested in position one, three of them had either a high leakage, a low (< 500 V) breakdown voltage, or a catastrophic failure. Comparison of measured results with simulation shows that the breakdown voltage of devices in positions 2 and 3 approach the theoretically predicted value for the utilized edge termination structure. The discrepancy between the magnitude of the leakage current prior to breakdown in simulation is due to lack of inclusion of additional trap states and tunneling mechanisms which contribute to excess current in the experimental devices. As the structure is not optimized, the electric field distribution within the defect free structure, shown in Fig. 7, reaches a peak value at the isolation implant edge, resulting in breakdown localized at that point. Thus, the reduction of breakdown below the ideal parallel-plane value for devices predicted by Raman to be outside of defective areas is a result of the consequences of edge termination design rather than the effects of the presence of a within a critical region.

Though measuring the shift in the E_2_ and A_1_ (LO) peaks in Raman spectra can predict variations in leakage current, on resistance, or high voltage breakdown, it does not correlate well with catastrophic failures in devices at lower voltages. For this, optical profilometry, in which many defects (in the form of bumps and pits) are observed, as seen in Fig. 4a and b, is found to better correlate. However, many of the defects are benign thus do not hinder the performance. Defects can be consistently observed at the corner and edge devices where the high crystal stress points occur indicating that the defects causing the high crystal stress propagate into the epitaxial layers and may hinder the quality as indicated by the histogram plot in Fig. 5. The regions with inhomogeneous conductivity do not consistently produce crystallographic defects thus it is not likely diminishing the quality of the epitaxial layers. The devices which catastrophically failed below 200 V reverse bias, do have indicators (highlighted with blue circles in Fig. S1 of the supplemental materials). Two of them have bumps in the implant isolation and edge termination region. They likely prevented the nitogen ion implantation from creating good edge termination and implant isolation, thus they had similar behavior to trench isolated diodes, which have leakage currents several order of magnitude larger^17^. The other two devices which catastrophically failed had pits greater than 1.5 µm deep as indicated in Fig. 4b. Since these pits are thicker than the p layer and possibly the drift layer, the current should travel through these weak points rather than the substrate, and are likely caused by defects on the substrate that didn’t allow epitaxial growth.

## Conclusion

In conclusion, arrays of vertical PiN diodes with planar JTE termination were fabricated on a inhomogeneous wafer with a regular patterning consisting of high crystal stress points and high resistivity points identified using Raman spectroscopy. The on resistance, leakage currents, and breakdown voltages were measured at multiple positions on the wafer relative to the high crystal stress points. It was determined that being closer to the stress points increases the reverse leakage current and increase the probability of failure at larger reverse bias voltages, while regions of inhomogeneous conductivity do not appear to affect the reverse bias properties, but slightly increase R_on_. Diodes off the crystal stress points consistently had breakdown voltages near the simulated value. Optical profilometry measurements reveal that the regions of high crystal stress can manifest themselves as changes in surface morphology, and regions with catastrophic failures typically have a bump or a pit present; however, many benign defects are detected as well, which indicates more sophisticated algorithms will be needed to effectively use optical profilometry to predict diode quality.. For example, machine learning with optical profilometry data as the input parameters could be trained using the diode IV data as the training set.

## Supplementary Information

Supplementary Information.

## Data Availability

All data and information nessesary for reproducing this work is in this published article and the supplementary information.
