# Supplementary material for: Optimizing performance and yield of vertical GaN diodes using wafer scale optical techniques
Source: Sci Rep. 2022 Jan 13;12:658. doi: 10.1038/s41598-021-04170-2 (PMC8758732; doi:10.1038/s41598-021-04170-2)
Supplement: Supplementary file 1 — Supplementary Information. [file 41598_2021_4170_MOESM1_ESM.docx]

Supplemental Materials for “Optimizing Performance and Yield of Vertical GaN Diodes using Wafer Scale Optical Techniques”

James C. Gallagher^1^, Mona A. Ebrish^2^, Matthew A. Porter^3^, Alan G. Jacobs^1^, Brendan. P. Gunning^4^, Robert. J. Kaplar^4^, Karl D. Hobart^1^, Travis J. Anderson^1^ Email:james.gallagher@nrl.navy.mil

1. U.S. Naval Research Laboratory, 4555 Overlook Ave SW, Washington, DC 20375
2. NRC Postdoc Fellow Residing at the U.S. Naval Research Laboratory
3. Naval Postgraduate School, 1 University Dr, Monterey, CA 93943
4. Sandia National Laboratories, PO Box 5800, MS 1086, Albuquerque, NM 87185

The supplemental materials contain data the complete data the optical profilometry (**Figure S1**), -200 V reverse leakage (**Figure S2**), and R_on_ (**Figure S3**).


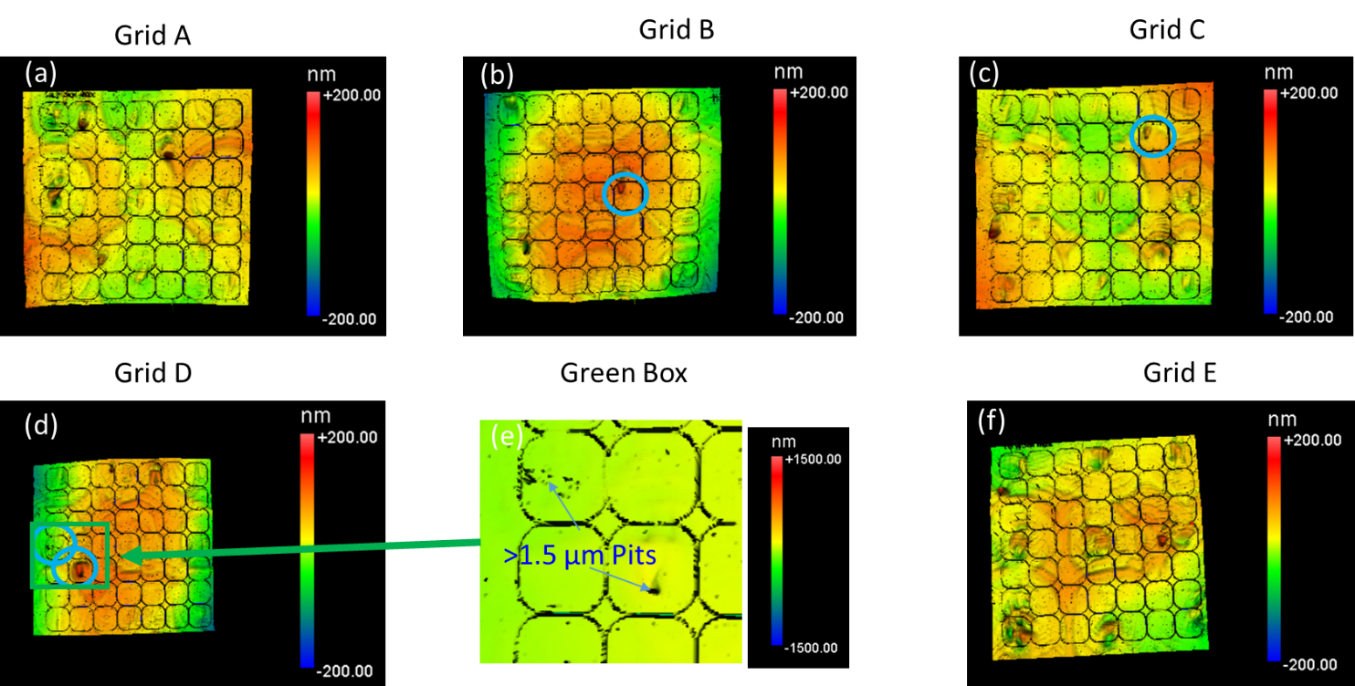


*Figure S1.* (a-d,f) Optical profilometry images of Grids A-E. No images exists for Grid F. The circled samples catastrophically failed. (e), an inset is showing the 1.5 µm pits causing catastrophic failure.


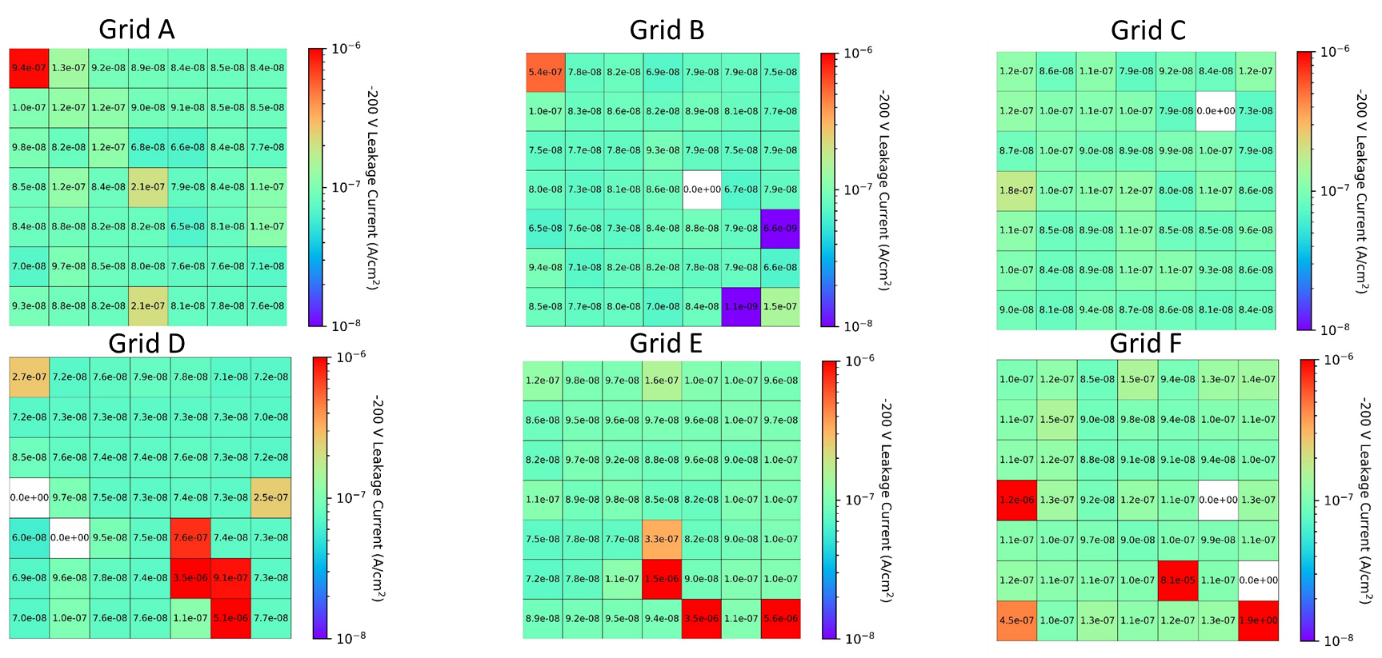


*Figure S2.* Reverse leakage current of 6, 7x7 reticles totaling 294 devices. The white devices catastrophically failed before reaching 200 V.


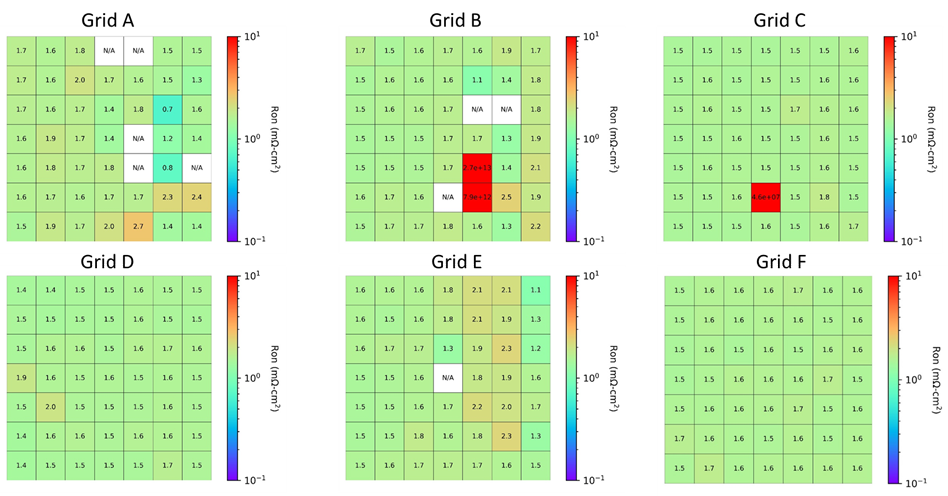


*Figure S3.* Plot of the on resistance (Ron) for the same devices as those in **Figure 5**. The white devices with “N/A” did not have enough data points in the on state region to get a good linear fit. These points were excluded when computing the data in **Table 1**.
